# HLA-associated protection of lymphocytes during influenza virus infection

**DOI:** 10.1186/s12985-020-01406-x

**Published:** 2020-08-24

**Authors:** Eliana E. Ochoa, Ruksana Huda, Steven F. Scheibel, Joan E. Nichols, David J. Mock, Nayef El-Daher, Frank M. Domurat, Norbert J. Roberts

**Affiliations:** 1 Division of Infectious Diseases, Department of Internal Medicine and the Department of Microbiology and Immunology, University of Texas Medical Branch, Galveston, TX USA; 2 Infectious Diseases Unit, University of Rochester School of Medicine, Rochester, NY USA; 3 Division of Infectious Diseases and Immunology, Department of Medicine, New York University School of Medicine, 462 First Ave, Room A619, New York, NY 10016 USA

**Keywords:** HLA, Human leukocyte antigen, Influenza virus, Human lymphocytes, Homozygosity, Monocytes/macrophages

## Abstract

**Background:**

Heterozygosity at HLA class I loci is generally considered beneficial for host defense. We report here an element of HLA class I homozygosity that may or may not help preserve its existence in populations but which could indicate a new avenue for antiviral research.

**Methods:**

Lymphocytes from serologically HLA-homozygous or -heterozygous donors were examined for synthesis of influenza virus proteins and RNA after exposure to virus as peripheral blood mononuclear cells. The virus-exposed lymphocytes were also examined for internalization of the virus after exposure, and for susceptibility to virus-specific cytotoxic T lymphocytes in comparison with virus-exposed monocytes/macrophages and unseparated peripheral blood mononuclear cells. Results were compared using two-tailed Fisher’s exact test.

**Results:**

Serologically-defined HLA-A2-homozygous lymphocytes, in contrast to heterozygous lymphocytes, did not synthesize detectable influenza virus RNA or protein after exposure to the virus. HLA-A2-homozygous lymphocytes, including both homozygous and heterozygous donors by genetic sequence subtyping, did internalize infectious virus but were not susceptible to lysis by autologous virus-specific cytotoxic T lymphocytes (“fratricide”). Similar intrinsic resistance to influenza virus infection was observed with HLA-A1- and HLA-A11-homozygous lymphocytes and with HLA-B-homozygous lymphocytes.

**Conclusions:**

A significant proportion of individuals within a population that is characterized by common expression of HLA class I alleles may possess lymphocytes that are not susceptible to influenza virus infection and thus to mutual virus-specific lysis. Further study may identify new approaches to limit influenza virus infection.

## Background

Heterozygosity at HLA class I loci is generally considered beneficial by assuring that individuals, and therefore the population, can respond to a wide variety of infectious challenges [1–3]. However, some alleles are so common, for example HLA-A1 and -A2 in Caucasians [4–6], that a subpopulation of up to 4–5% would be homozygous by serological typing or, even in some populations for HLA-A2, by 6-digit high resolution sequence-based typing [7]. This might reflect selection due to recurrent or predominant infectious threats to the population [8–10], but that is not established. Influenza virus is such a recurrent (seasonal) and predominant (pandemic) threat [11, 12] but, when carefully examined, more of the T lymphocyte immune response, such as interferon production or cytotoxic T cell activity, is restricted to the more heterogeneous HLA-B alleles rather than to HLA-A alleles [13]. The importance of HLA expression to defense against the infection is potentially further indicated by the observation that both influenza A and influenza B virus downregulate expression of Class I HLA [14].

The current studies were undertaken in response to some surprising HLA-related results in studies of the requirement for monocyte/macrophage participation for influenza virus infection of human lymphocytes [15, 16]. Those studies showed that influenza virus infection of human lymphocytes occurs in the immune cell cluster of the developing antiviral response. Although peripheral blood mononuclear cells (PBMC) from many donors had been studied, and had shown such a requirement for lymphocyte infection, lymphocytes from two individuals did not synthesize viral proteins despite being exposed in the presence of monocytes/macrophages. Our PBMC donor pool of slightly more than 100 individuals had previously been serologically tissue-typed, and we determined that the two donors shared homozygous expression of HLA-A2. We then initiated the current studies, specifically examining the infection of PBMC using cells from all of the HLA-A homozygous donors in our donor pool, as well as the single available HLA-B homozygous donor, with concurrent analyses of cells from heterozygous donors. We report here a unique, and surprising, potentially protective element for human lymphocytes associated with HLA class I serological homozygosity that may suggest new antiviral approaches to influenza virus infection even if it does not help preserve and ensure the existence of HLA homozygosity in populations.

## Methods

### Cell sources and culture conditions

PBMC were obtained from the peripheral blood of healthy adult volunteers by Ficoll-Hypaque sedimentation [17]. Informed oral consent was obtained from all cell donors and the studies were approved by the Institutional Review Boards for human subject research of the University of Rochester and the University of Texas Medical Branch. Identifying numbers were assigned to the five different HLA-A2 (numbers 1–5) and the three HLA-A1 (numbers 6–8) homozygous donors to indicate, via the figure legends, that all of the homozygous donors could contribute to the data presented in the figures and tables, showing a consistency for the reported observations. All of the donors had no illnesses or vaccinations in the 2 months prior to blood donation, but all were assumed to have had past natural influenza virus infections. In line with that assumption, we [18] and others [19] have shown in earlier studies that adult leukocyte donors have class I–restricted immunity against influenza A viruses that is characterized by the persistence, after clearance of infection, of circulating influenza antigen-specific human T cells.

Donors were typed for HLA class I and class II antigens, using established serological techniques for class I antigens and commercially available typing reagents. For a subset of donors, cellular DNA was extracted using the Easy DNA kit for genomic DNA isolation (Invitrogen, Carlsbad, CA) and HLA-A2 subtyping was performed using the Dynal HLA-A2 PCR sequence specific primers (SSP) subtyping kit (Lake Success, NY) [20]. All experiments used concomitant assays of autologous cell preparations. The PBMC were cultured at 37 °C in medium 199 (M199) with 10% heat-inactivated fetal calf serum (FCS) except during one-hour exposures to infectious influenza virus when they were suspended in serum-free medium [21, 22].

### Collection of purified monocyte-macrophage and total lymphocyte populations

Purified lymphocytes (> 99.5% purity and > 97% viability) were obtained by elutriation [15, 23, 24] using a Beckman J2–21 centrifuge with a JE-6 elutriator rotor and Multiperplex pump with fine velocity control (LKB Instruments). Purified monocytes/macrophages were obtained by adherence separation [21, 22] and were equivalent in responses to monocytes/macrophages obtained by elutriation. Purity of cells was always confirmed by immunofluorescent staining and flow cytometry analysis [25].

Limited PBMC were donated by the HLA-B-homozygous donor. Therefore, T lymphocytes were separated from PBMC using Dynal T cell Negative Isolation Kit Ver II (Invitrogen, CA) rather than by elutriation.

### Exposure to infectious influenza virus

The PBMC or purified subpopulations of PBMC were exposed to infectious influenza A/Marton/43 (H1N1) at a multiplicity of infection (MOI) of three unless noted otherwise [21, 22]. In a subset of experiments, PBMC and purified lymphocytes were exposed to FITC-labeled influenza virus [26]. The cells were analyzed for synthesis and expression of viral proteins by pulse labeling and immunofluorescent staining, respectively [15, 27]. Limiting dilutions of monocytes/macrophages were tested in each assay and showed that if a minimal and undetectable contamination of lymphocyte preparations by monocytes/macrophages were present, it could not account for results of assays of lymphocyte preparations.

In another subset of experiments, sham-exposed and influenza virus-exposed PBMC, monocytes-macrophages, or lymphocytes were treated with neuraminidase (Clostridium-derived, Type V, Sigma) which removes surface-bound virus [28, 29]. Flow cytometric analyses of cells exposed to FITC-labeled virus and treated with neuraminidase showed that all detectable virus was removed from the cell surface [26].

### Flow cytometry analyses of cell phenotype and virus uptake

Phenotypes of separated populations were determined by direct immunofluorescent staining and flow cytometry (FACScan; Becton Dickinson, Mountain View, CA) [27, 30], identifying CD3^+^ T lymphocytes (anti-Leu-4) and CD14^+^ monocytes/macrophages (anti-Leu-M1) (Becton Dickinson). Cells that were exposed to FITC-labeled virus were analyzed for bound versus internalized virus using resonance energy transfer techniques described previously [26, 31]. For each flow analysis, 10,000 cells were examined.

### Analyses of viral protein and RNA synthesis

Lysates of pulse-labeled cells were analyzed by SDS-polyacrylamide gel electrophoresis (SDS-PAGE) and autoradiography [15, 32]. Immunoprecipitation was performed using NIAID Reference Reagent antibodies specific for the H1 (Reagent #V-314-511-157) viral hemagglutinin (HA), the N1 (#V-308-513-157) neuraminidase (NA), and influenza A matrix (M) protein (#V-306-501-157), as well as a murine monoclonal anti-nucleoprotein antibody [33].

Total cellular RNA was obtained from sham-exposed and influenza virus-exposed PBMC at varying times (0–24 h) after exposure and analyzed using Northern blots [34, 35]. A cRNA strand complementary to the 1413 nucleotides of the influenza A/WSN neuraminidase (N1) positive strand (mRNA and template RNA), a kind gift from Dr. Louis Markoff, NIAID, was used for the analyses.

For both viral protein and RNA synthesis assays, photographic reproductions of gels were prepared with relatively reduced exposure of lanes containing lysates of monocytes-macrophages, which were intense relative to lanes containing lysates of lymphocytes.

### Infectious focus assay for influenza virus infection

HLA-A2-homozygous lymphocytes were exposed to the virus in the presence of monocytes/macrophages for 1 h before purification and treatment with neuraminidase. The lymphocytes were then layered over uninfected monocytes/macrophages for 1 h before removal from the monocytes/macrophages. The lysates of the monocytes/macrophages and lymphocytes purified after exposure to the virus, as well as the monocytes/macrophages that were exposed to the lymphocytes, were each collected after a subsequent 5 h of incubation. At the same time as they were layered over the uninfected monocytes/macrophages, lymphocyte samples were also layered over MDCK cell monolayers which were overlaid subsequently with 0.6% agarose, incubated at 37 °C for 48 h, then fixed and stained with methylene blue to facilitate plaque quantification. Based on previous studies, one plaque was assumed to be caused by one infected cell [36].

### Assays of cytotoxic T lymphocyte (CTL) activity

PBMC were infected with influenza A/Marton/43 (H1N1) and cultured for 6 days to generate CTL (effector cells) [37]. Nonadherent cells (CTL) were collected and counted using trypan blue to determine viability. Influenza virus-infected target cells were prepared using the inducing strain of virus, A/Marton/43 (H1N1), as well as influenza A/Scotland/840/74 (H3N2) and B/Singapore/3/64 viruses. The target cell cultures were incubated for 1 h and left unseparated, or separated into purified macrophage and purified lymphocyte/lymphoblast populations, using washing and elutriation as above [15, 24]. Autologous CTL activity was then examined using the purified macrophages, purified lymphocytes/lymphoblasts, and unseparated PBMC as parallel targets in a standard 4-h ^51^Cr-release assay [38].

### Quantitative real-time PCR analysis

Differential expressions of the influenza A/Marton/43 hemagglutinin (HA) and neuraminidase (NA) genes [39] were confirmed by quantitative real-time PCR (qRT-PCR) on an ABI PRISM 7000 sequence detection system (Applied Biosystems, Foster City, CA, USA). RNA was isolated using RNeasy kit (Qiagen, CA). Following DNAse digestion (Invitrogen, CA), cDNAs were prepared from all replicate RNA samples (Access RT-PCR system, Promega, WI) and subjected to qRT-PCR experimentation. For qRT-PCR, primer sequences were designed based on Primer3 (http://frodo.wi.mit.edu/cgi-bin/primer3/primer3_www.cgi) and DNAMan 6.0 (http://lynon.com) softwares: HA (AF494248.1) - Forward 5′-CCC AAACACAACACAACCAG-3′ and Reverse 5′-GCAAGGTCCAGTAAT AGTTCATCC-3′; NA (AY122326.1) -Forward 5′-GTC TGA ATG TGCCTGCGTAA-3′ and Reverse 5′-CAG TTG CCT TTT TCC ATC TTTG − 3′. All primers were synthesized by Sigma-Genosys (http://www.sigma-genosys.com/utmb/). Random primers and SYBR green PCR Master Mix were purchased from Roche (Indianapolis, IN) and Applied Biosystems (Foster City, CA), respectively. An annealing temperature of 60 °C was used to amplify cDNAs. For each different primer set, a no template control (NTC) was used to confirm amplification of specific PCR product in the post-amplification dissociation curve. After normalization to 18S rRNA (Ambion, TX) amplification, differential gene expression was measured by following the Cycle-threshold (ΔCt) method described in the manufacturer’s protocol. PCR products (~ 300 base pairs) were further verified by gel electrophoresis and sequencing.

### Statistical analysis

Comparison of the responses of HLA-homozygous and HLA-heterozygous lymphocytes to exposure to influenza virus were performed using two-tailed Fisher’s exact test.

## Results

### Influenza virus protein and RNA are synthesized by HLA-A2-heterozygous but not HLA-A2-homozygous lymphocytes

We previously showed that human monocyte/macrophage and lymphocyte interaction after exposure to influenza virus, including H1N1, H2N2 and H3N2 strains, is required for synthesis of viral proteins by lymphocytes [15, 16]. In recent studies, we have used cells from individuals who were on a panel of slightly more than 100 subjects who had been serologically HLA-typed. The studies reported here included cells from every HLA-homozygous individual (10 in total) on that panel.

**Fig. 1:**
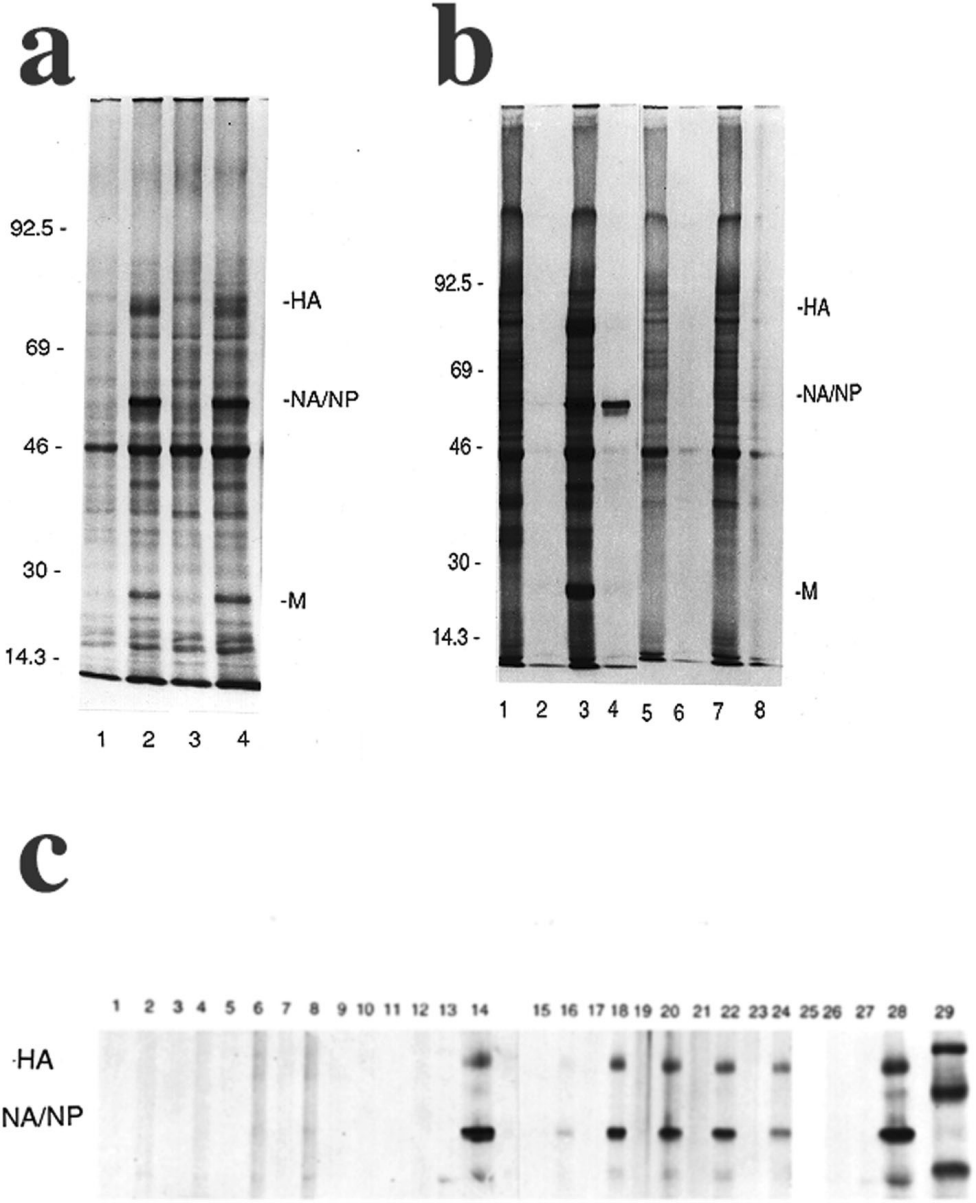
Protein Synthesis by Influenza Virus (IAV)-exposed Human PBMC. These representative images show results using cells from HLA-A2-homozygous donor number 1 in part b and HLA-A2-homozygous donor number 2 in part c. **a** Autoradiograms (monocytes/macrophages, lanes 1, 2; purified lymphocytes, lanes 3, 4) show representative protein synthesis results using PBMC from an HLA-A2-.heterozygous donor after sham-exposure or exposure of PBMC to IAV at an MOI = 10. Odd-numbered lanes show lysates of sham-exposed cells, and even-numbered lanes show lysates of IAV-exposed cells. HA = hemagglutinin, NA/NP = neuraminidase and nucleoprotein, which co-migrate, and M = the matrix protein. Numbers show positions of standard proteins having the indicated Mr. × 10^− 3^. **b** Autoradiograms show representative protein synthesis results using PBMC from an HLA-A2-homozygous donor. After sham-exposure (lanes 1, 2 and 5, 6) or exposure to IAV as PBMC at an MOI = 10 (lanes 3, 4 and 7, 8), purified monocytes/macrophages (lanes 1–4) and lymphocytes (lanes 5–8) were obtained and pulse-labeled and analyzed. Odd-numbered lanes show total cell lysates, and even-numbered lanes show lysates immunoprecipitated with mouse monoclonal anti-NP antibody. **c** Autoradiograms show cell lysates from an HLA-A2-homozygous donor (lanes 1–14) and an HLA-A1,2 donor (lanes 15–28) that were immunoprecipitated using mouse monoclonal anti-NP antibody and polyclonal anti-HA and anti-NA antibodies. After sham-exposure (odd-numbered lanes) or exposure to IAV (even-numbered lanes) as unseparated PBMC, purified lymphocytes and monocytes/macrophages were obtained, pulse-labeled and collected. Lymphocytes were pulse-labeled 0–2 h (lanes 1, 2 and 15, 16), 2–4 h (lanes 3, 4 and 17, 18), 4–6 h (lanes 5, 6 and 19, 20), 6–8 h (lanes 7, 8 and 21, 22), 8–10 h (lanes 9, 10 and 23, 24), and 22–24 h (lanes 11, 12 and 25, 26) after exposure. Monocytes/macrophages (lanes 13, 14 and 27, 28) were pulse-labeled 4–6 h after exposure. Lane 25 is blank (lysate not available). Lane 29 shows positions of standard Mr. proteins

Both virus-exposed purified monocytes-macrophages and lymphocytes from HLA-A2-heterozygous donors synthesized viral hemagglutinin, neuraminidase, matrix protein, and nucleoprotein, detected by pulse-labeling the cells 4–6 h after exposure to the virus (Fig. 1a). The data are representative of consistent results using cells from HLA-A2-heterozygous donors analyzed concurrently with cells from homozygous donors. In fact, virus-exposed monocytes/macrophages from all donors tested in these and earlier studies synthesized viral proteins. However, virus-exposed lymphocytes from HLA-A2-homozygous donors showed no detectable viral protein synthesis (Fig. 1b). Immunoprecipitation with murine monoclonal antibody to influenza A nucleoprotein [33] and polyclonal anti-hemagglutinin, anti-neuraminidase, and anti-matrix protein antisera confirmed the absence of detectable viral protein synthesis by either virus-exposed resting lymphocytes or by PHA-stimulated lymphocytes/lymphoblasts (Fig. 1b) from all five HLA-A2-homozygous donors that were identified and tested.

The time for pulse-labeling the cells in the above experiments was chosen, based on earlier kinetic experiments [15], for ability to detect maximal viral protein synthesis. However, the lack of viral protein synthesis by HLA-A2-homozygous lymphocytes could have reflected significantly altered kinetics rather than the absence or severe reduction of synthesis of viral proteins. Therefore, in further experiments, analysis of virus-directed protein synthesis by HLA-A2-heterozygous lymphocytes was compared to that by lymphocytes from HLA-A2-homozygous donors at times ranging from 0 to 2 to 22–24 h after exposure to virus. Figure 1c presents the results of such a sequential time-course analysis, showing radiolabeled and immunoprecipitated lysates from the lymphocytes. Immunoprecipitation was again performed using the antibodies noted above. Maximal viral protein synthesis was demonstrated in lysates collected from 4 to 8 h after exposure to virus only in the HLA-A2-heterozygous lymphocytes in every time-course experiment. In some time-course experiments, synthesis of viral proteins was less evident (than shown in Fig. 1c) using lysates collected beyond 8 h after exposure. Identical results were obtained using lymphocytes from heterozygous donors not expressing HLA-A2. In contrast to these results, pulse-labeling of HLA-A2-homozygous lymphocytes followed by immunoprecipitation failed to demonstrate virus-directed protein synthesis at any time point examined for all five donors (Fig. 1c). As noted above and also shown in Fig. 1c, monocytes/macrophages from all donors synthesized influenza virus proteins. The results shown in each section of Fig. 1 are representative of five experiments using cells from different heterozygous and homozygous donors tested concurrently.

**Fig. 2:**
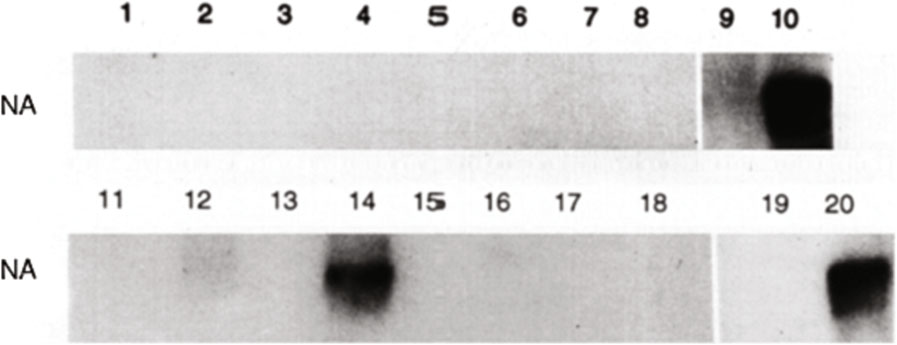
Influenza Virus (IAV) Neuraminidase (NA) RNA is Synthesized by HLA-A2- heterozygous (HTZ) but not HLA-A2-homozygous (HMZ) Lymphocytes. This representative image shows results using cells from HLA-A2-homozygous donor number 3. Autoradiograms of Northern blots show cell lysates from an HLA-A2-HMZ donor (lanes 1–10) and an HLA-A1,2 donor (lanes 11–20) obtained after sham-exposure (odd-numbered lanes) or exposure to IAV (even-numbered lanes). After exposure as unseparated PBMC, purified lymphocytes were obtained and lysates were collected after 2 h (lanes 1, 2 and 11, 12), 4 h (lanes 3, 4 and 13, 14), 8 h (lanes 5, 6 and 15, 16), and 24 h (lanes 7, 8 and 17, 18). Lysates of purified monocytes/macrophages (lanes 9, 10 and 19, 20) were collected 4 h after exposure. Lysates were probed for positive strand NA as described in the Methods. The original autoradiogram of the HLA-A1,2 donor’s lysates also showed a faint NA signal in lane 16 as well as 12 and 14

Further analysis of PBMC from both HLA-A2-homozygous and HLA-A2-heterozygous individuals was performed using Northern blots for the detection of positive strand viral RNA indicating transcription of the viral neuraminidase gene product (Fig. 2). Uninfected control monocytes/macrophages were negative but, as expected from the above results measuring viral protein synthesis, viral RNA was present in virus-exposed monocytes/macrophages from both HLA-A2-homozygous and HLA-A2-heterozygous individuals at 3–4 h after exposure. Virus-exposed lymphocytes obtained from HLA-A2-heterozygous donors also showed detectable viral antigenomic RNA. In contrast, no viral RNA was detected in lymphocytes from any of the five HLA-A2-homozygous donors at any time examined after exposure to virus (Fig. 2), suggesting that some form of pre-transcriptional block to replication was the basis for lack of evidence of viral protein synthesis in HLA-A2-homozygous lymphocytes.

Both the HLA-A2-homozygous and the HLA-A2-heterozygous donors used for Fig. 2 are different from the donors used for Fig. 1b and Fig. 1c in order to illustrate the consistent association of homozygous expression of HLA-A2 with absent synthesis of influenza virus gene products by lymphocytes. Lymphocytes from all five HLA-A2-homozygous donors showed no detectable synthesis of influenza virus RNA or proteins even when exposed in the presence of monocytes/macrophages, the latter cells shown in previous studies to be required for the infection of human lymphocytes [15, 16].

### Lymphocyte resistance to influenza virus infection is associated with HLA class I serologic but not genotypic homozygosity

We determined whether our five serologically-defined homozygous HLA-A2 donors (3 male and 2 female) were homozygous at the level of genetic sequence subtyping. The donors included both genotypically homozygous (e.g., HLA-A*02:03-A*02:03) and heterozygous (e.g., HLA-A*02:03-A*02:11) individuals: two were homozygous and three were heterozygous by genetic sequence subtyping. The donors were heterozygous for all the other HLA class I determinants. As described above, the data showed that none of the serologically homozygous HLA-A2 lymphocytes synthesized viral RNA or proteins after internalization of the virus whether the cells were genotypically homozygous or heterozygous.

### Influenza virus is internalized by HLA-A2-homozygous lymphocytes

Lymphocytes were obtained from pairs of HLA-A2-homozygous and HLA-A2-heterozygous donors and exposed to FITC-labeled influenza virus in the presence of autologous monocytes/macrophages. The monocytes/macrophages and lymphocytes within the mixed cultures were identified and analyzed using flow cytometry, with addition of ethidium bromide to distinguish internalized from external cell-bound virus using fluorescence resonance energy transfer [26]. As expected, internalization of virus was demonstrated by HLA-A2-homozygous and HLA-A2-heterozygous monocytes/macrophages when they were exposed in the presence of autologous lymphocytes (Table 1). Most importantly, internalization of the virus was demonstrated by lymphocytes, when they were exposed to the virus in the presence of monocytes/macrophages, whether the lymphocytes were HLA-A2-homozygous or HLA-A2-heterozygous (Table 1).

**Table 1:** Internalization of FITC-labeled influenza virus by monocytes/macrophages and lymphocytes

Experiment	HLA-A determinants of donor^a^	Percent positive cells^b^	
		Monocytes/macrophages^c^	Lymphocytes
1	2,28	59.75	3.01
	2,-	61.47	4.94
2	1,11	20.58	3.78
	2,-	17.79	3.16
3	1,2	24.58	1.14
	2,-	21.54	1.26

^a^Cells from three different HLA-A2-homozygous donors (numbers 1, 4 and 5) were used

^b^Cells containing FITC-labeled influenza virus-derived green fluorescence after addition of ethidium bromide to quench FITC-derived green fluorescence external to the cells. 10^4^ cells were analyzed for each determination

^c^Monocytes/macrophages were identified by forward and side light scatter as well as expression of CD14

**Fig. 3:**
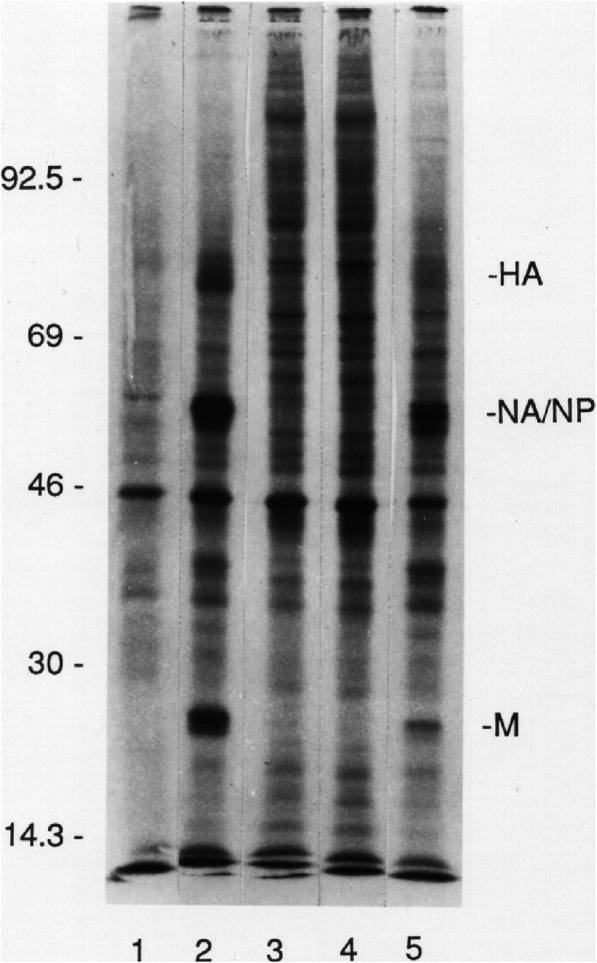
Influenza Virus (IAV)-infected HLA-A2-homozygous Lymphocytes Serve as Infectious Foci for Uninfected Monocytes-macrophages. This representative image shows results using cells from HLA-A2-homozygous donor number 2. Autoradiograms show representative protein synthesis results using PBMC from an HLA-A2-homozygous donor. After sham-exposure (lanes 1 and 3) or exposure to IAV at an MOI = 10 (lanes 2 and 4), purified macrophages (lanes 1 and 2) and lymphocytes (lanes 3 and 4) were obtained and pulse-labeled. Additional aliquots of virus-exposed lymphocytes, after separation from macrophages by elutriation, were treated with neuraminidase, washed, and layered over additional aliquots of autologous control macrophages. After 1 h, the latter macrophages were extensively washed free of lymphocytes, pulse-labeled, and analyzed (lane 5)

**Fig. 4:**
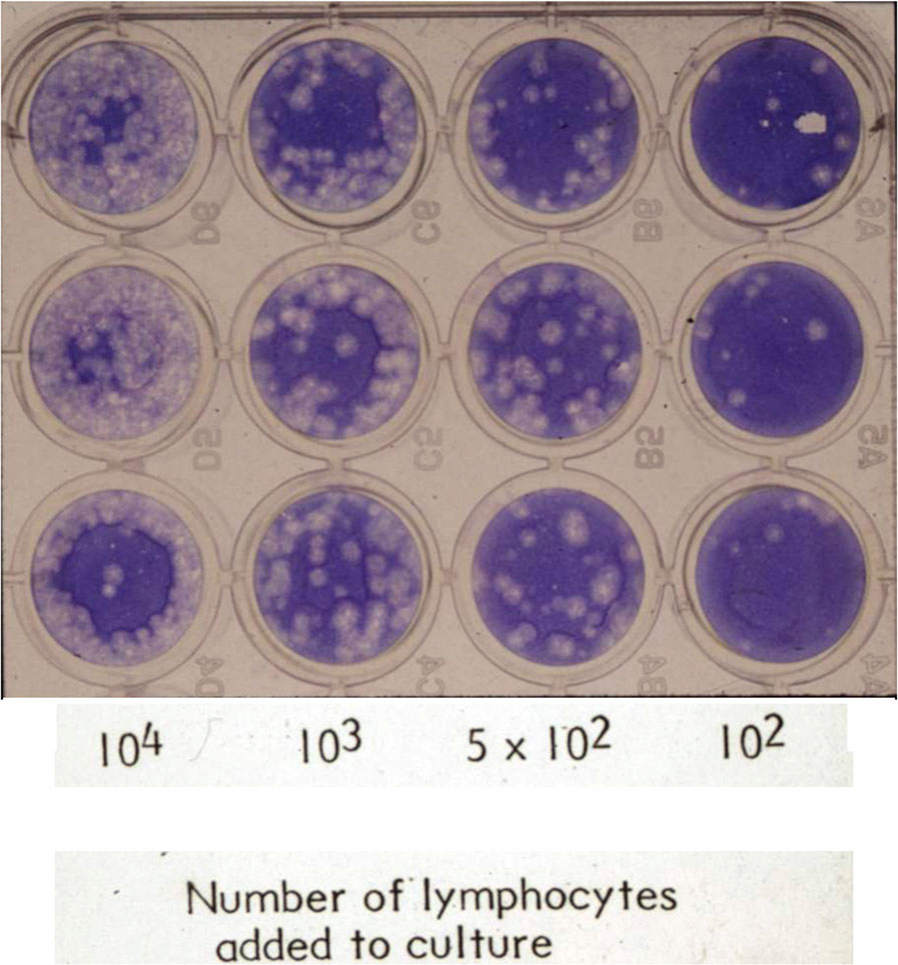
Influenza Virus (IAV)-infected Neuraminidase-treated HLA-A2-homozygous Lymphocytes Serve as Infectious Foci for MDCK Cells. This representative image shows results using cells from HLA-A2-homozygous donor number 2. Lymphocytes were layered over MDCK cell monolayers which were overlaid subsequently with 0.6% agarose, incubated at 37 °C for 48 h, then fixed and stained with methylene blue to facilitate plaque quantification. Based on previous studies, one plaque was assumed to be caused by one infected cell

Modified infectious focus assays were then used in several experiments to detect infectious virus present in HLA-A2-homozygous lymphocytes after exposure to virus in the presence of monocytes-macrophages. Lymphocytes were layered over influenza virus-exposed monocytes-macrophages which had been treated with neuraminidase to remove any potential extracellular virus [26]. The lymphocytes were then collected, treated with neuraminidase, and layered over autologous control macrophages. Evidence of viral protein synthesis by each of the cell populations was then sought, including by the latter cells after removal of the lymphocytes. The results (Fig. 3) indicated that, after exposure in the presence of macrophages, HLA-A2-homozygous lymphocytes contained infectious influenza virus, and were able to infect co-cultured autologous control macrophages. In addition, the virus-exposed, neuraminidase-treated HLA-A2-homozygous lymphocytes were able to serve as infectious foci for the fully virus-permissive Madin-Darby canine kidney (MDCK) cell line (Fig. 4), with subsequent production of infectious progeny virus by that cell line.

### The susceptibilities of HLA-A2-heterozygous and HLA-A2-homozygous lymphocytes to mutual cell-cell killing differ

Further studies were performed to determine whether there was a correlation between decreased or absent synthesis of influenza viral proteins in lymphocytes of HLA-A2-homozygous donors and the results when such cells were used as targets in an autologous cytotoxic T lymphocyte (CTL) assay. Such an analysis could also be considered more sensitive for evidence of infection since major CTL targets like the viral nucleoprotein can lead to lysis despite an inability to detect them on the cell surface by other methods [40, 41]. Other investigators have shown that both CD4+ and CD8+ cloned effector cells are themselves susceptible to immune-mediated killing, by induction of apoptosis, when incubated with their specific antigens, commonly demonstrated by addition of their peptide epitopes [42–44], but such killing is likely to occur as well when they are themselves infected and naturally present such epitopes. CTL can be as susceptible to peptide-mediated lysis as conventional target cells, and are not refractory to the lytic mechanisms of other CTL even though they avoid destruction by their own lytic mediators when delivering the lethal hit [44]. Mutual CTL-CTL killing has been termed “fratricide” [45].

**Fig. 5:**
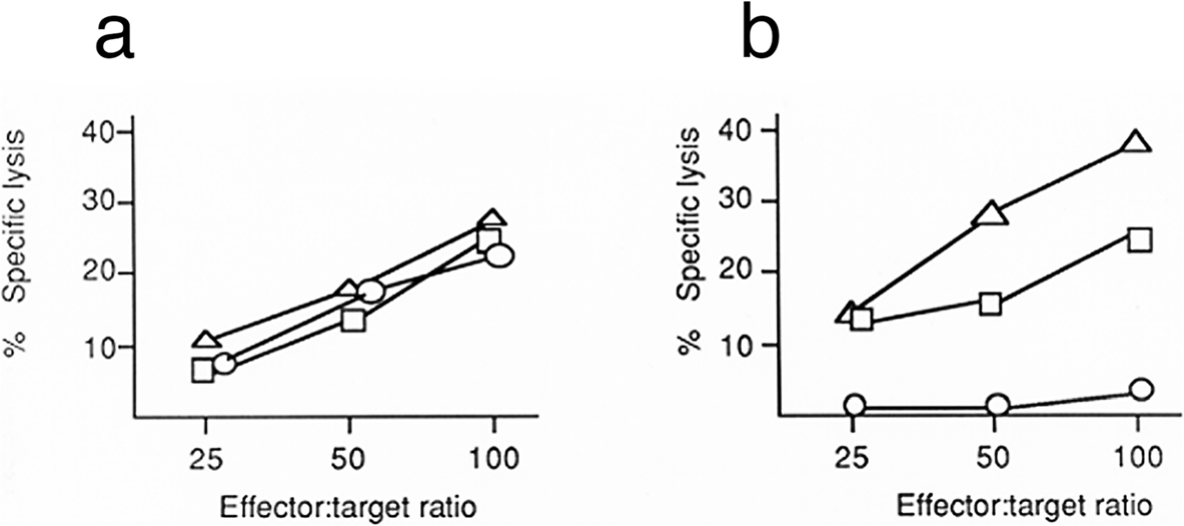
Influenza Virus (IAV)-specific Cytotoxicity is Related to HLA-A Determinants and Target Cell Populations. This representative image shows results using cells from HLA-A2-homozygous donor number 4. Cytotoxic T lymphocyte (CTL) activity was measured as specific lysis of autologous targets consisting of monocytes/macrophages (Δ), or lymphocytes/lymphoblasts (○), or unseparated PBMC (□). Results show virus-specific lysis of autologous target cell populations infected with the strain of virus used to induce CTL (A/Marton/43 H1N1). The graphs show CTL activity and autologous target cell susceptibilities for PBMC and subpopulations from (**a**) a heterozygous (A1,11) individual and (**b**) a homozygous (A2, −) individual. Results are representative of three experiments using PBMC from different heterozygous and homozygous donors tested concurrently

Data are shown in Fig. 5 which are representative of consistent results using cells from our HLA-A2-heterozygous and non-HLA-A2 heterozygous donors as both autologous effector cells and target cells. There was clear virus-specific lysis of all three heterozygous autologous target cell populations: unseparated peripheral blood mononuclear cells (PBMC), purified monocytes/macrophages, and purified lymphocytes/lymphoblasts (Fig. 5a). In contrast, there was no detectable lysis of virus-exposed autologous lymphocytes/lymphoblasts from HLA-A2-homozygous donors (Fig. 5b). These results were not due to an inability to generate CTL which were specific for influenza virus, as indicated by lysis of virus-exposed HLA-A2-homozygous monocytes/macrophages and unseparated PBMC (containing monocytes/macrophages). The CTL activity was influenza virus-specific since the CTL lysed autologous PBMC infected with the inducing influenza strain (H1N1) or, to a lesser extent, an alternate (H3N2) strain of influenza A, but did not lyse uninfected PBMC, or PBMC infected with influenza B/Singapore (data not shown in figure). Similar results were obtained using cells from all three HLA-A2-homozygous donors tested, and cells from HLA-A2-heterozygous donors tested concurrently. Such results provide further evidence of the lack of infection of the homozygous lymphocytes.

### Influenza virus protein is synthesized by HLA-A1, A11, and HLA-B heterozygous but not homozygous lymphocytes

**Fig. 6:**
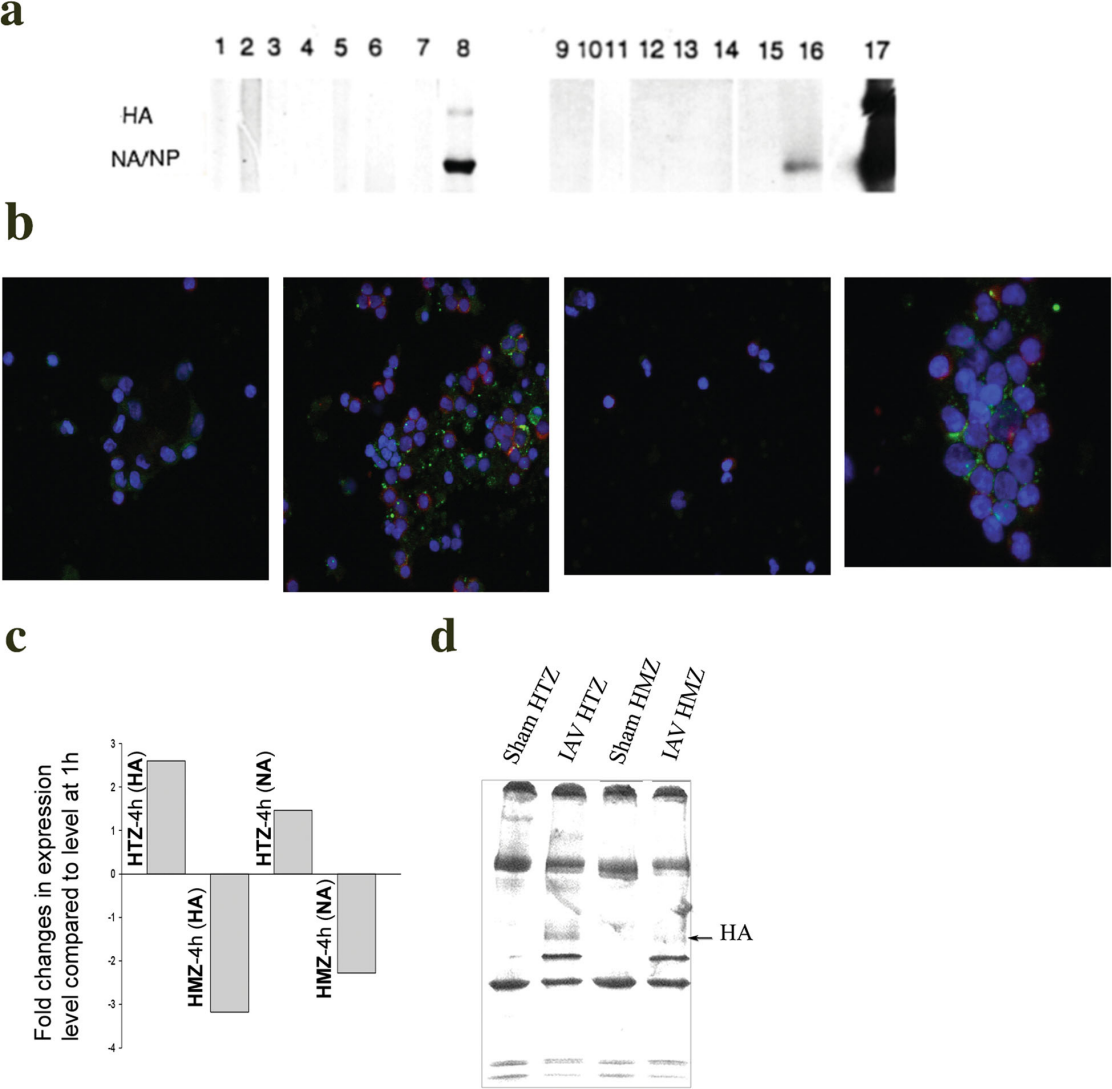
Influenza Virus (IAV) Proteins Are Synthesized by Virus-exposed Human HLA-A1, HLA-A11, and HLA-B Heterozygous (HTZ) but not Homozygous (HMZ) Lymphocytes. This representative image shows results using cells from HLA-A1-homozygous donor number 6 in part a. **a** Autoradiograms show representative results of viral protein synthesis using PBMC from an HLA-A1-HMZ donor (lanes 1–8), and results using PBMC from the HLA-A11-HMZ donor (lanes 9–16). After sham-exposure (odd-numbered lanes) or exposure to IAV (even-numbered lanes), purified monocytes/macrophages (lanes 7,8 and 15,16) and lymphocytes (lanes 1–6 and 9–14) were obtained and pulse-labeled and analyzed. Lymphocytes were pulse-labeled 2–4 h (lanes 1, 2 and 9, 10), 4–6 h (lanes 3, 4 and 11, 12), and 6–8 h (lanes 5, 6 and 13, 14) hours after exposure. Cell lysates immunoprecipitated with anti-influenza hemagglutinin (HA), neuraminidase (NA) and nucleoprotein (NP) antibodies are shown. Lane 17 shows positions of standard Mr. proteins. Data are representative of consistent results using cells from the three available HLA-A1-homozygous donors and the single HLA-A11-homozygous donor. **b** Confocal images of sham-exposed and IAV-FITC-exposed lymphocytes. Fields from left show sham-exposed HLA-A-HTZ/−B-HTZ lymphocytes; IAV-FITC-exposed HLA-A-HTZ/−B-HTZ lymphocytes; sham-exposed HLA-A-HTZ/−B-HMZ lymphocytes; and IAV-FITC-exposed HLA-A-HTZ/−B-HMZ lymphocytes, respectively. **c** Real time PCR differential fold-change in expression of HA and NA at 4 h post-infection compared to the level at 1 h in lymphocytes of HLA-B-HTZ and HLA-B-HMZ individuals. qPCR values represent mean of two independent experiments using replicate samples (*n* = 3). 18S rRNA primers were used as internal control for normalization of data. **d** Analysis of viral HA synthesis by T-lymphocytes from HLA-B-HTZ and -HMZ individuals. Lymphocytes were isolated from infected PBMCs at 4 h post-infection, pulse-labeled for 2 h, and cell lysates were immunoprecipitated with anti-HA antibody and autoradiographed

Studies were next performed to determine whether the above-described lack of detectable viral protein synthesis by purified lymphocytes was specific to the homozygous expression of HLA-A2, or would be evident also in studies using lymphocytes from donors with homozygous expression of other HLA alleles. We were able to obtain cells from the three donors in our panel with homozygous expression of HLA-A1, and one donor with homozygous expression of HLA-A11 (an Asian donor; a subject from a population in which HLA-A11 is common). Lymphocytes were exposed to virus in the presence of monocytes/macrophages, purified, and pulse-labeled at varying times after exposure as in the above experiments. All monocytes/macrophages synthesized viral proteins after exposure in these studies. In all experiments, lymphocytes from the donors with homozygous expression of the HLA-A alleles showed no detectable synthesis of influenza virus proteins (Fig. 6a). In respective concurrently conducted assays, lymphocytes from donors with heterozygous expression of HLA-A1 or HLA-A11 showed synthesis of viral proteins. The data are representative of consistent results using cells from the three HLA-A1-homozygous donors. Further studies with FITC-labeled influenza virus (IAV-FITC) showed that the HLA-A1- and HLA-A11-homozygous lymphocytes internalized the virus just as did HLA-A2-homozygous or any HLA-A-heterozygous lymphocytes in the earlier or concurrent experiments, respectively (Table 2).

**Table 2:** Internalization of FITC-labeled influenza virus by monocytes/macrophages and lymphocytes

Experiment	HLA-A determinants of donor	Percent positive cells^a^	
		Monocytes/macrophages^b^	Lymphocytes
1	1,2	10.89	4.55
	1,-	13.36	2.56
2	3,11	30.89	7.09
	11,-	21.15	3.76

^a^Cells containing FITC-labeled influenza virus-derived green fluorescence after addition of ethidium bromide to quench FITC-derived green fluorescence external to the cells. 10^4^ cells were analyzed for each determination. This table shows results using cells from HLA-A1-homozygous donor number 7

^b^Monocytes/macrophages were identified by forward and side light scatter as well as expression of CD14

We then had the opportunity to study PBMC from a single donor whose cells were homozygous for HLA-B44 but heterozygous for all other HLA alleles, in comparison with HLA-B44-heterozygous cells tested concurrently. Cells from both donors took up the IAV-FITC (Fig. 6b). Since limited cells could be obtained from that donor, we used real time PCR (Fig. 6c) and 4–6 h post-exposure pulsing with autoradiography of immunoprecipitated cell lysates (Fig. 6d) to assess the viral infection. Lymphocytes were isolated from virus-infected PBMCs at 1 h and 4 h post-infection. Both HA and NA RNA at 4 h post-infection sharply decreased (2–3-fold) or degraded in homozygous but not in heterozygous lymphocytes, and HA and NA RNA continued to increase in the heterozygous lymphocytes (Fig. 6c). Lymphocytes from the HLA-B-homozygous donor failed to synthesize influenza virus glycoproteins, just as observed with HLA-A-homozygous lymphocytes, whereas HLA-B-heterozygous lymphocytes showed clear new synthesis of the viral proteins such as the HA (Fig. 6d).

### Analysis of the different responses of HLA class I-homozygous and HLA Class I-heterozygous lymphocytes to influenza virus

The different responses of the lymphocytes from HLA Class I homozygous and heterozygous donors were analyzed (Table 3) and showed highly statistically significant differences (*P* < 0.0001) in response to exposure to the virus.

**Table 3:** Analysis of lymphocyte responses to influenza virus exposure

	VP-positive^a^ lymphocytes	VP-negative lymphocytes	Total
HLA Class I heterozygous lymphocytes	10	0	10
HLA Class I homozygous lymphocytes	0	10	10
Total	10	10	20

^a^*VP* Virus proteins

*P* < 0.0001 by two-tailed Fisher’s exact test

## Discussion

Most individuals survive seasonal and even pandemic influenza and recover completely with establishment of homotypic immunity due to cell-mediated responses to the challenge. Murine models have been used to demonstrate rapid and substantial recruitment of PBMC to the lung after influenza virus challenge [46–49]. These recruited cells play important roles in defense against and recovery from the virus infection [50, 51]. Such PBMC are themselves susceptible to infection by the virus [15, 16] and, although infection of the PBMC is abortive, their infection may be an important component of overall influenza pathogenesis. Ultimately, constitutional resistance to influenza virus and similar pathogens is likely to be under complex polygenic control, such that simple division of a population into discrete susceptible and resistant groups should not be expected [52].

It is well recognized that CD8+ CTL contribute greatly to the clearance of virus-infected cells and promote recovery from influenza virus infection [53]. The current studies indicate that HLA-A2-homozygous CTL are generated and as effective as HLA-A2-heterozygous CTL but are not susceptible to fratricide. Only 1–4% of influenza virus-exposed lymphocytes, but as many as 40–70% of monocytes-macrophages, become infected as measured by uptake of FITC-labeled virus or by infectious focus assays [16, 26]. The standard CTL assay using PBMC target cells may thus obscure even such profound differences in lymphocyte infection as those demonstrated for lymphocytes that are homozygous for HLA-A2, since such cells would represent a small minority of the potential infected target cells.

HLA polymorphism has generally been thought to be selected by the pressure of infectious challenges [1–3, 54]. With the diverse number of pathogens that may be encountered, it is reasonable to ask why certain populations have preserved very common expression of alleles, such as HLA-A1 or HLA-A2 in Caucasians. As mentioned above, this might reflect selection due to recurrent or predominant infectious threats to the population [8–10]. We are not aware of any clinical evidence that HLA class I-homozygous individuals are protected from severe or lethal influenza virus infection. There have been many studies that examine gene variants that contribute to enhanced susceptibility or resistance to various viral diseases. For influenza virus, however, reports have generally been limited to identification of variants that are associated with severe disease or complications of the infection [55–57]. With regard to HLA specifically and susceptibility in influenza virus infection, analysis of targeting efficiency has been correlated with human T cell response magnitude and with mortality [58]. In those studies, a population-based analysis found that the carriage frequencies of the alleles with the lowest targeting efficiencies, such as A*24, were associated with pandemic A/H1N1 influenza virus (pH1N1) mortality; such alleles are common in certain indigenous populations in which increased pH1N1 morbidity has been reported.

In studies perhaps most relevant to our in vitro observations, Falfán-Valencia and colleagues examined the frequencies of HLA Class I alleles and haplotypes in regard to genetic susceptibility to the pH1N1 virus, using 6-digit high resolution sequence-based typing [7]. In infected subjects (138 individuals with documented infection) and concurrent control subjects (225 asymptomatic healthy contacts), all Mexican mestizos by ethnicity, there was no difference between the groups in the percent of subjects with the equivalent of homozygous serological typing (A*02:xx:xx-A*02:xx:xx). Thus, 3.985% of the infected subjects and 4.000% of the control subjects had the equivalent of serological homozygosity (Ramcés Falfán-Valencia, personal communication). Those studies did show HLA-related risk; for example, A*68:01:01 was exclusively present in the infected group [7]. Thus, data to support protection are elusive. It would be helpful to establish a DNA bank from patients with influenza virus infection to support studies of human genetic variation and its association with the pathogenicity of influenza, as recommended by Zhang and colleagues [59].

An extensive impact of influenza virus infection on cellular pathways, demonstrated using HeLa cells, is mediated by both replication-dependent and, at early stages, replication-independent events [60], with both upregulation and downregulation of a large number of genes. Global human leukocyte responses to influenza virus are likely to be equally complex. A thorough literature review in 2008 proposed a systems-based list of approximately 100 candidate genes for future study of the genetic basis of influenza disease and immunity in humans, citing evidence of their potential role in the pathogenesis of the infection [59].

The current studies have certain limitations. They were limited in the number of subjects who could donate PBMC for the experiments, and the panel of donors that we could access had been HLA-serotyped rather than typed by genetic sequence. However, it should be noted that the ten individuals (5 HLA-A2-homozygous, 3 HLA-A1-homozygous, 1 HLA-A11-homozygous, and 1 HLA-B44-homozygous) included every one of the HLA class I homozygous subjects from our typed donor panel, and each was studied concomitantly with a heterozygous subject from the panel. When the five serologically HLA-A2-homozygous individuals were subsequently genotyped, they included both homozygous and heterozygous individuals by 4-digit genotyping. Representative autoradiograms and other analyses were provided in the results, but we note again that results were consistent for all of the homozygous lymphocytes which were all tested. In fact, the representative data provided for each figure, or section thereof, and for the tables were from different homozygous donors in order to demonstrate the consistency of the findings. None of the HLA class I homozygous lymphocytes synthesized influenza proteins even though they internalized the virus just as did heterozygous lymphocytes after exposure in the presence of monocytes/macrophages. Another limitation is that the current studies cannot establish that the protection of HLA-homozygous lymphocytes from infection works at a population level to ensure a proportion of HLA-homozygous donors in the population who are relatively protected from influenza virus infection, or perhaps other challenges, which might relate to natural selection for the species.

Influenza virus binds to the majority of both human monocytes/macrophages and lymphocytes [26]. The virus is internalized by a large percentage of monocytes/macrophages but by only a small percentage of lymphocytes [26]. In the current and previous studies, autoradiograms of virus-exposed purified monocytes/macrophages showed vigorous viral protein synthesis by cells from all donors [15, 16]. The data thus suggested that the absence of viral protein synthesis in lymphocytes of HLA-A2-homozygous donors was not due to monocyte/macrophage resistance to viral infection. However, previous studies also showed that monocytes/macrophages are required for influenza virus infection of human lymphocytes [15, 16], and such results raised the possibility that HLA-A2-homozygous monocytes/macrophages, once infected, are unable to transfer the virus to lymphocytes during cell-cell interaction. The studies using exposure of the cells to FITC-labeled influenza virus showed that the lymphocytes did internalize the virus after exposure in the presence of the monocytes/macrophages. The current studies used a well-established H1N1 clinical isolate strain of influenza virus [39]. However, whenever we have extended our studies to other strains of influenza virus, we have found that results such as presented using the H1N1 strain are replicated. For example, the requirement of monocytes/macrophages for lymphocyte infection was evident for strains from all three subtypes of human IAV (H1N1, H2N2 and H3N2) [15].

Lymphocytes are not the primary target cells for influenza virus and they only become infected when recruited to the respiratory tract to participate in immune defense. We do not know whether respiratory epithelial cells, the desired target for the virus, would show a similar effect of HLA homozygosity. It may be that such resistance to the infection is limited to lymphocytes since monocytes/macrophages from homozygous donors are infected by influenza virus to an extent equivalent to that of cells from heterozygous donors.

It is puzzling that A1- and A2-homozygous lymphocytes show resistance to influenza virus infection whereas A1-A2-heterozygous lymphocytes do not. It is also puzzling that the lymphocyte phenotype is defined serologically but not genotypically. It is clear that the HLA-homozygous lymphocyte resistance to infection occurs at a stage after entry of the virus into the cell. The IFITM family of interferon-stimulated genes inhibit an early viral entry step [61] and might deserve future investigation related to our observations. Genome-wide RNA interference screening using lung epithelial (A549) cells has identified ten human host proteins involved in post-entry steps of influenza virus replication [62], including nuclear import components, proteases, and the calcium/calmodulin-dependent protein kinase (CaM kinase) IIb (CAMK2B). It is not known whether the same proteins would be implicated in post-entry infections of human lymphocytes. However, one or more of these proteins, such as CAMK2B, involved in transcriptional regulation, might be implicated in future studies to determine the mechanism for HLA-homozygous lymphocyte resistance to the infection.

## Conclusions

The results of the current studies delineate a unique HLA-associated lymphocyte defense against a major pathogen, influenza virus, that produces recurring epidemic and pandemic challenges. Further investigation offers an opportunity to elucidate a mechanism for cellular resistance to influenza virus infection that may provide direction for novel antiviral development. We hope that other investigators with access to larger panels of HLA-typed donors are able to extend these studies.

## Data Availability

The datasets used and/or analysed during the current study are available from the corresponding author on reasonable request.

## Abbreviations

HA: Hemagglutinin
HMZ: Homozygous
HTZ: Heterozygous
IAV: Influenza A virus
M: Matrix protein
NA: Neuraminidase
NP: Nucleoprotein
VP: Viral proteins
